# Disruption of the endocannabinoid system is prominent in lung adenocarcinoma and associated with poor patient survival

**DOI:** 10.1186/2049-3002-2-S1-P76

**Published:** 2014-05-28

**Authors:** Kelsie Thu, Roland Hubaux, Stephen Lam, Wan Lam

**Affiliations:** 1 Integrative Oncology, BC Cancer Agency Research Centre, Vancouver, BC, Canada

## Background

The endocannabinoid system (ECS) is comprised of cannabinoid receptors, lipid messengers called endocannabinoids, and the enzymes that metabolize them. It modulates numerous processes including the central nervous and immune systems as well as cell signaling pathways important in normal function and diseases such as cancer. Many studies have demonstrated anti-tumour effects of synthetic and endogenous cannabinoids in cancer, emphasizing the potential for ECS modulators as anti-cancer therapies. We believe that ECS deregulation may contribute to the malignant phenotype of lung cancer cells by reducing anti-tumour endocannabinoid levels and consequently increasing levels of arachidonic acid, which can promote tumour growth. We sought to determine whether the ECS is disrupted in clinical lung tumours and whether disruption has prognostic implications.

## Materials and methods

We hypothesize that recurrent downregulation of cannabinoid receptors (CNR1, CNR2) or upregulation of metabolizing enzymes (FAAH, FAAH2, MGLL) occurs in lung tumours to prevent the anti-tumor effects of endocannabinoids. We assessed the gene expression, copy number, and DNA methylation status of these ECS components in two independent lung cancer cohorts with genomic profiles for patient matched lung adenocarcinoma and non-malignant tissues (BC Cancer Agency (BCCA) and The Cancer Genome Atlas (TCGA)). We also investigated associations between ECS disruption and patient outcome.

## Results

The ECS was recurrently disrupted in both lung adenocarcinoma cohorts; 78% and 82% of tumours in the BCCA (n=83) and TCGA (n=57) cohorts, respectively, harboured gene expression changes consistent with our hypothesis in at least one of the five components assessed. Underexpression of CNR1 and overexpression of FAAHs were the most prominent alterations observed, and were associated with copy losses and gains, respectively. Frequent promoter hypomethylation affecting CNRs and FAAH was also observed but was only associated with concordant overexpression for FAAH. Survival analysis revealed ECS disruption, specifically CNR1 underexpression and/or FAAH overexpression, was significantly associated with poor survival (Mantel-Cox p-value = 0.0128) in the BCCA cohort. A similar non-significant trend was observed in the TCGA cohort (Mantel-Cox p-value = 0.0561).

## Conclusions

Components of the ECS undergo frequent genetic changes which may compromise the function of this system in lung cancer cells. The recurrent nature of ECS disruption and its association with poor prognosis suggests deregulation is selected for in lung adenocarcinoma. We believe ECS disruption status may predict sensitivity of lung cancer cells to the anti-tumour effects of ECS targeting agents and plan to investigate this in future work.

